# The Impact of a Single Dose of a Polyphenol-Rich Seaweed Extract on Postprandial Glycaemic Control in Healthy Adults: A Randomised Cross-Over Trial

**DOI:** 10.3390/nu10030270

**Published:** 2018-02-27

**Authors:** Margaret Murray, Aimee L. Dordevic, Lisa Ryan, Maxine P. Bonham

**Affiliations:** 1 Department of Nutrition, Dietetics and Food, Monash University, Level 1, 264 Ferntree Gully Road, Notting Hill, VIC 3168, Australia; Margaret.murray@monash.edu (M.M.); aimee.dordevic@monash.edu (A.L.D.); 2 Department of Natural Sciences, Galway-Mayo Institute of Technology, Dublin Road, Galway H91 T8NW, Ireland; lisa.ryan@gmit.ie

**Keywords:** functional food, phlorotannin, polyphenol, diabetes, glucose, insulin, ethnicity

## Abstract

This study investigated the impact of a polyphenol-rich seaweed extract on postprandial glycaemia in healthy adults, and, as a secondary outcome, the influence of ethnicity on these outcomes. Thirty-eight volunteers (26 non-Asian, 12 Asian) aged 19 to 56 years participated in this double-blind, placebo-controlled, randomised cross-over trial. Participants each consumed a low (500 mg), and high (2000 mg) dose of the polyphenol-rich brown seaweed (*Fucus vesiculosus*) extract, as well as a cellulose placebo (2000 mg), 30 min prior to 50 g of available carbohydrate from white bread. Postprandial blood glucose and plasma insulin concentrations were measured over two hours (fasting, 15, 30, 45, 60, 90, and 120 min) from a finger prick blood sample. Data were analysed using a repeated measures analysis of variance. Compared with the placebo, neither dose had a lowering effect on postprandial glucose or insulin responses. However, individuals of an Asian background experienced consistently elevated plasma insulin responses, assessed using an incremental area under the curve, compared with non-Asian participants, irrespective of supplement (*p* = 0.016). These results suggest an increased risk of insulin resistance among Asian populations, compared with non-Asian, and that measurement of blood glucose levels alone may be insufficient to diagnose diabetes risk in this population.

## 1. Introduction

Type 2 diabetes mellitus (T2DM) is a chronic disease, caused when the body is unable to effectively use insulin to maintain blood glucose levels. T2DM now affects hundreds of millions of adults worldwide [1], but at particularly high risk of T2DM are those of an Asian background who have adopted a Western lifestyle [2,3]. Postprandial hyperglycaemia (prolonged high blood glucose level following a meal) is common in T2DM, and over time leads to serious diabetes-related complications, as well as playing a role in metabolic syndrome and increasing the risk of cardiovascular diseases [1,4,5,6]. This is particularly relevant to Asian populations, where there is evidence to indicate elevated glycaemic responses following carbohydrate foods, compared with European populations [2]. Prevention and management of hyperglycaemia is primarily through lifestyle modification, with diet and exercise strategies and weight management [7]. However, long-term weight maintenance is challenging [8,9]. Therefore, it is important to consider other strategies to prevent or manage hyperglycaemia, such as the identification of bioactive food components, which are able to modulate glucose metabolism and thus reduce the risk of chronic disease [10]. 

Epidemiological evidence from Asian countries indicates that a diet rich in seaweed (marine macroalgae) is associated with longevity and a decreased risk of chronic diseases, such as T2DM [11,12]. Marine macroalgae, particularly brown algae, are a rich source of bioactive compounds, including lipids (eicosapentaenoic acid), polysaccharides (fucoidan), minerals, and phytochemicals, with potential to improve health [13]. Recently, there has been increased interest in the potential health effects of bioactive polyphenols from marine macroalgae, particularly the main sub-group unique to marine algae called phlorotannins, which are highly hydrophilic polymers of phloroglucinol units (1,3,5-trihydroxybenzene), ranging in size from 120 Da to 650 kDa [4,10,14]. There is accumulating evidence, from both in vitro and animal models, that macroalgal polyphenols may assist in the prevention or management of T2DM through inhibiting carbohydrate digestive enzymes α-amylase and α-glucosidase, increasing glucose uptake by skeletal muscle tissue, and inhibiting hepatic glucose output [4,10,14,15]. 

Alpha-glucosidase inhibitors are a class of drugs used to reduce postprandial hyperglycaemia by delaying carbohydrate digestion and absorption of glucose into the blood, the same proposed action as polyphenols [16]. However, these drugs have known side effects, and often are only prescribed for patients with a more advanced diabetic state. Plant polyphenols (from fruits and berries) successfully lower postprandial blood glucose responses, through delaying carbohydrate breakdown and glucose absorption at an intestinal level [17]. Macroalgal polyphenols may provide an alternative that can be used to help control postprandial glycaemia and prevent the development or progression of T2DM. Polyphenols from a number of seaweed species, including *Ascophyllum nodosum*, *Ecklonia stolonifera*, *Fucus vesiculosus* and *Ishige okamurae*, have demonstrated inhibition of α-amylase and α-glucosidase in vitro [18,19,20,21,22]. Confirmation of this effect has been observed in mouse and rat models, with blunted postprandial blood glucose levels observed following supplementation [19,20,21,22]. However, to date only two studies have examined the effect of macroalgal polyphenols on postprandial glycaemic responses in humans. One study of 63 adults with elevated fasting plasma glucose found a reduction in 2-h postprandial blood glucose concentration in their intervention group compared with the placebo [23]. The other saw no differences in the postprandial blood glucose response, but did observe a reduction in the plasma insulin incremental area under the curve (iAUC) in 23 healthy adults following intervention, compared with the placebo [24]. There is the potential that the differences in these results may be somewhat due to differing ethnic backgrounds of the study populations, as the first study was carried out in Korea, whereas the second was in Canada [2,25]. 

This double-blind, placebo-controlled, randomised cross-over trial explored the effectiveness of two different doses of a polyphenol-rich extract sourced from *Fucus vesiculosus* at lowering postprandial glycaemic response, in a healthy adult population. A novel aspect of this research is the examination of the impact of ethnicity on the effect of a polyphenol-rich seaweed extract on glycaemic control. The examination of a multi-cultural population, including a high proportion of individuals from an Asian background, has enabled comparison between those of Asian and non-Asian backgrounds. These comparisons are important, as the majority of algal polyphenol-related research to date has been carried out in Asia, with findings therefore limited to clinical trials conducted on Asian populations [26]. Undertaking this study in population groups outside of Asia, as well as with Asian populations who have migrated away from their country of birth, will contribute to the growing literature [26]. These findings have particular relevance given the growing population of Asian individuals adopting Western lifestyles [27] and their increased risk of T2DM [2,3]. 

## 2. Materials and Methods

### 2.1. Trial Design

This study was a double-blind, placebo-controlled, randomised cross-over trial conducted between May and December 2016 in Melbourne, Australia, and reported according to the CONSORT 2010 checklist. It was registered with the Australian New Zealand Clinical Trials Registry (ANZCTR), registration number ACTRN12616000126415p (available at http://www.anzctr.org.au/ACTRN12616000126415p.aspx). Ethics approval for the project was granted by the Monash University Human Research Ethics Committee (MUHREC), approval number CF16/53-2016000019. All procedures were carried out according to the Declaration of Helsinki. Written informed consent was obtained from all participants. 

### 2.2. Participants

Participants were recruited from the public via advertising flyers, personal contacts, snowballing, mail-outs, and by contacting participants from local databases. Male and female volunteers, aged 18 to 65 years, with a body mass index (BMI) from 18.5 to 28 kg/m^2^, a fasting blood glucose level below 5.5 mmol/L, and blood pressure within the normal range (systolic blood pressure ≤ 140 mmHg, diastolic blood pressure ≤ 90 mmHg) were eligible to participate. Participants were excluded if they had any gastrointestinal issues that might affect the absorption and intestinal actions of the polyphenols; were taking medication for blood sugar control; were taking other natural health products known to impact on polyphenols (e.g., fish oil), were breastfeeding or pregnant; had liver or thyroid issues; had hypertension; had undergone recent major surgery; consumed more than four standard drinks per day, or 9 standard drinks per week; were a cigarette smoker; or had an implanted cardiac defibrillator. Participants were also excluded if they were unable to consume any of the study food due to dietary requirements. For all menstruating female participants, testing sessions were avoided in the seven-day period prior to the beginning of menstruation. 

Power analyses were performed prior to the start of the study, in order to identify an appropriate sample size. Based on data reported in the literature [24,28], 36 individuals were required to detect a 40-unit difference in blood glucose and 2500-unit difference in plasma insulin iAUCs between the supplements and placebo at a significance level of 0.05 and power of 80%. To allow for a participant withdrawal rate of 10%, the recruitment target was set at 40 participants.

### 2.3. Randomisation and Blinding

Computer-generated random numbers were used to determine the sequence in which participants received their supplements. All supplements were encapsulated in identical brown capsules and labelled with a corresponding letter (A, B, or C) to conceal the identity of the supplement that participants were given on each testing occasion. The participants and investigator carrying out data collection and analysis (MM) were blinded to which supplement was consumed on each occasion. The generation and allocation of the random supplement order to participants was carried out by a separate investigator, who was not involved with data collection (MB). Only the researchers (HW-A, HI, and ES) who administered the supplement doses were aware of which supplement participants received at each crossover. Participant enrolment, data collection, and statistical analysis of results were carried out by the investigator (MM), who remained blinded to supplement allocation until analyses were completed. 

### 2.4. Test Products

The intervention product was Maritech^®^ Synergy, a powdered extract from the macroalga *Fucus vesiculosus*, certified by the company to contain 28% polyphenols and 67% fucoidan (a complex carbohydrate) (Marinova Pty Ltd., Cambridge, TAS, Australia). The powder was encapsulated in opaque size 0 capsules (The Melbourne Food Depot, East Brunswick, VIC, Australia) for ease of consumption and dosing. Doses of 500 mg (containing 140 mg polyphenols) and 2000 mg (containing 560 mg polyphenols) were each administered on a single occasion prior to a carbohydrate load and postprandial blood tests. These doses were selected as they are similar to doses used in previous human trials (144 mg [29] and 690 mg [23]), which have shown significant improvements in other biochemical markers (e.g., LDL cholesterol, fasting glucose). Two different doses were selected to elicit whether there was a dose-response relationship. The placebo was Medisca^®^ Cellulose, NF (Microcrystalline) (MEDISCA Australia Pty Ltd., Mascot, NSW, Australia) administered in a dose of 2000 mg to match the total mass of the high-dose intervention product. A cellulose fibre placebo was selected to account for the 67% complex carbohydrate content of the intervention supplement.

### 2.5. Procedure

Interested participants were initially screened via telephone interview. Those who met the inclusion criteria were then screened in person at the university research facility to ensure that their fasting blood glucose level was less than 5.5 mmol/L, that their BMI was between 18.5 and 28 kg/m^2^, and that their blood pressure was within the normal range (systolic blood pressure ≤ 140 mmHg, diastolic blood pressure ≤ 90 mmHg). Eligible participants were then invited into the study and randomised to an intervention order.

Participants attended three testing visits at the university research facility, over which they received a single dose of the high and low doses of the test product and the placebo in a randomised order. Participants were provided with a pre-prepared standardised evening meal (pasta with tomato-based sauce; 3425 kJ, 122 g carbohydrate, 3.3 g fat, 20.4 g protein) to consume between 7 and 9 pm the night before each testing session, then were asked to fast from 9 pm onwards, with the exclusion of water. Participants were also asked to avoid a list of foods that are naturally high in polyphenols and refrain from strenuous exercise for 24 h prior to each testing session.

On the mornings of each testing session, two fasting (≥10 h) finger prick blood samples were taken to provide fasting levels of blood glucose (−45 and −35 min) and insulin (−45 min only). At −30 min participants were administered either the low (500 mg) or high (2000 mg) dose of Maritech^®^ Synergy or placebo. Thirty minutes later, at time 0, participants consumed 50g available carbohydrate in the form of white bread (108 g bread). Over the following two hours, finger prick blood samples were taken at pre-determined intervals to measure blood glucose (15, 30, 45, 60, 90, 120 min) and insulin (30, 60, 90, 120 min). Blood sampling was conducted for two hours following carbohydrate consumption, in accordance with standard oral glucose tolerance test protocol [30].

Glucose concentrations were assessed instantaneously from the whole blood finger prick samples using the HemoCue Glucose 201 RT System (Radiometer Pacific Pty Ltd., Mount Waverley, VIC, Australia), according to standard procedures. To measure plasma insulin 200 µL capillary blood samples were collected from the finger prick using Safe-T-Fill™ Capillary Blood Collection GK Systems containing EDTA anti-coagulant (item no. 077001, Kabe Labortechnik GmbH, Nümbrecht, Germany). Samples were centrifuged for 15 min at 4 °C at 1.3 RCF (serial no. 5703BI110739, Eppendorf AG, Hamburg, Germany). The plasma was aliquoted and stored at −80 °C until analysis. This process was repeated 3 times in a crossover manner, so that all participants received the two supplement doses and placebo. Each treatment was separated by a washout period of at least 5 days.

### 2.6. Outcome Measures

#### 2.6.1. Biochemical Measurements

Plasma insulin concentrations were measured using Millipore ELISA Kits for Human Insulin (Cat. # EZHI-14K and EZHI-14BK, Merck Millipore, Bayswater, VIC, Australia), with absorbance measured using the Rayto Microplate reader (450 nm wavelength, RT-2100C, Abacus ALS, Meadowbrook, QLD, Australia), according to kit instructions. Sample volumes of 20 µL were pipetted into wells, with each sample assessed in duplicate. The lowest level of insulin detectable by this assay was 1.0 µU/mL, so any concentration values that were below this were designated 1.0 µU/mL. The range of the assay was up to 200 µU/mL. Samples that read above this value were diluted 2:1, using assay buffer as a diluent, and re-run. Across all plates, the mean coefficient of variation was 8.6% (SD 13.8). Insulin units were converted to pmol/L prior to statistical analysis.

#### 2.6.2. Anthropometric Data

Anthropometric measurements were taken at the screening session. Height was measured using the Harpenden Stadiometer (Holtain Ltd., Crymych, UK), with shoes and socks removed. Weight and body composition (% fat mass, % fat free mass, visceral fat (L)) were measured using the SECA mBCA 515 medical body composition analyser (Seca GmbH & Co. KG, Hamburg, Germany), with shoes and socks removed and participants in light clothing. The SECA mBCA 515 has been validated for use in an ethnically diverse population [31]. A waist circumference measure was also taken over light clothing or bare skin at the narrowest point around the torso [32].

#### 2.6.3. Intolerance Symptoms

A questionnaire, adapted from the intolerance symptoms questionnaire used by Paradis et al. [24] was completed by participants 24 h after ingestion of the supplements, to assess the occurrence and intensity of any side effects. Participants indicated whether side effects were absent, or of mild, moderate, or severe intensity (scored as 0, 1, 2 or 3, respectively). Side effects included in the questionnaire related to headache, energy levels, appetite, gastrointestinal symptoms, unusual pain or sensations, cardiac palpitations, balance disorders, and depression/anxiety.

#### 2.6.4. Diaries and Questionnaires

Food intake data were collected using a three-day food diary (over two weekdays and one weekend day) to establish participants’ usual dietary intake, and assessed using FoodWorks 8 (Xyris Software (Australia) Pty Ltd., Spring Hill, QLD, Australia). A food frequency questionnaire (FFQ), adapted from a British FFQ designed to assess polyphenol intake [33], was used to estimate participants’ usual polyphenol intake, based on data from Phenol-Explorer 3.6 Database on polyphenol content in foods [34], and the USDA Database for the Flavonoid Content of Selected Foods, Release 3.1 (December 2013) [35]. Physical activity habits were assessed using the International Physical Activity Questionnaire short form [36].

### 2.7. Quantification of Soluble Phlorotannins

The quantification of total soluble phlorotannins in the extract was performed using an adapted Folin-Ciocalteu method [37], with phloroglucinol dihydrate used as standard (item no.: P38005, Sigma-Aldrich, Castle Hill, NSW, Australia), to confirm the reported polyphenol content. The sample was dissolved in 10 mL distilled water, and then diluted to reach concentrations of 25, 50, and 100 µg/mL. Quantification was carried out by pipetting 2 mL of distilled water (blank), phloroglucinol standards (5, 10, 15, 20, 30, 50, and 100 µg/mL), and the sample solutions into sequential vials. Then 500 µL of Folin-Ciocalteu reagent (item no.: F9252, Sigma-Aldrich, Castle Hill, NSW, Australia) was added and allowed to stand for 5 min. This was followed by the addition of 1500 µL of 7.5% *w*/*w* sodium carbonate solution and 4000 µL of distilled water. The reaction was then incubated in the dark at room temperature for two hours. The solutions were analysed in quartz cuvettes using a spectrophotometer at 765 nm, and absorbance values were recorded. All samples, standards, and blanks were run in triplicate.

### 2.8. Statistical Analysis

All results were assessed for normality using the Shapiro-Wilk test for normality. Where results were non-parametric the data were log transformed, using the natural log, and parametric tests were applied. Summary statistics for parametric data are reported as mean (standard deviation), and for non-parametric data are reported as median (interquartile range). The level of significance was accepted at *p* ≤ 0.05. Analyses were performed using the Statistical Package for Social Sciences (SPSS) version 22.0 (SPSS Inc., Chicago, IL, USA). Postprandial responses for blood glucose and insulin were assessed by iAUC, time to peak, and peak blood concentration. 

A repeated measures analysis of variance (ANOVA) was used to determine any significant differences among the three treatments for fasting concentrations, postprandial iAUC, and peak concentrations of blood glucose and plasma insulin. Supplement sequence, age, gender, and % fat mass were added to the ANOVA as covariates for postprandial iAUC and peak concentration analyses. Mixed between-within-subjects ANOVAs were conducted, to determine whether there was a difference in glucose and insulin (iAUC and peak concentration) between participants from Asian and non-Asian backgrounds across the three treatment types. Where there was a difference between the ethnic groups, independent *t*-tests were used to identify with which treatment type(s) these differences were observed. Due to the number of additional comparisons planned (three per ANOVA), a Bonferroni adjustment was made to the alpha level for the independent *t*-tests, resulting in significance at *p* < 0.017 [38]. Differences between the groups for intolerance symptoms were assessed using the Friedman’s test. Time to peak data is presented as frequency (%).

## 3. Results

### 3.1. Participants

**Figure 1:**
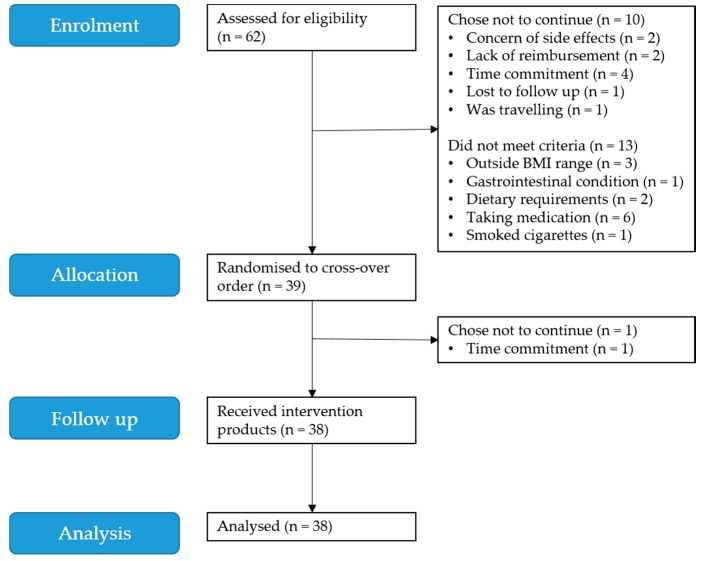
Participant flow diagram.

Baseline participant characteristics are presented in Table 1. From April to December 2016, 39 individuals were recruited into the study. One participant withdrew after commencing the study due to an inability to commit the time required, resulting in a total of 38 participants (nine men and 29 women) who completed the protocol (Figure 1). Participants were aged from 19 to 56 years (median 23 years), with a BMI ranging from 18.9 to 28.3 kg/m^2^ (median 21.9 kg/m^2^). Twelve (32%) participants were of an Asian background (nine female, three male) and 26 (68%) of a non-Asian background (20 female, six male). Asian participants included those who self-identified as Chinese (*n* = 9), Indian (*n* = 2), and Indonesian (*n* = 1). Non-Asian participants included those who self-identified as white Australian (*n* = 20), British (*n* = 1), Polish (*n* = 1), Persian (*n* = 1), Turkish (*n* = 1), Italian (*n* = 1), and Greek (*n* = 1). Asian participants had significantly more visceral adipose tissue compared with non-Asian participants (*p* < 0.001), despite there being no differences between the ethnicities for total fat mass as a percentage of body weight (*p* = 0.659). All participants had a fasting blood glucose level below 5.5 mmol/L (mean (SD): 4.2 (0.4) mmol/L) and blood pressure readings within the normal range (systolic blood pressure ≤ 140, and diastolic blood pressure ≤ 90). 

### 3.2. Postprandial Blood Glucose

**Figure 2:**
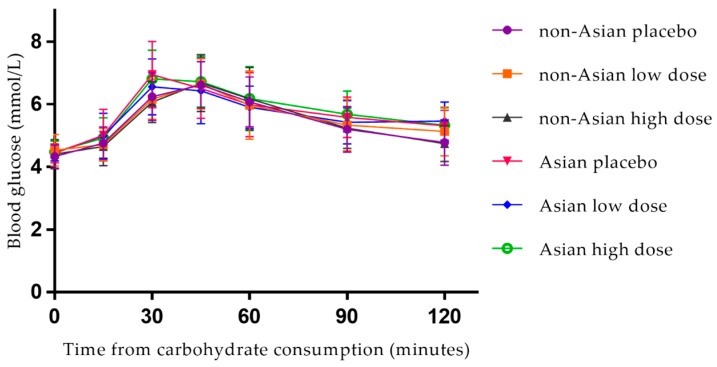
Postprandial blood glucose response for non-Asian participants (*n* = 26) and Asian participants (*n* = 12), following consumption of 50 g available carbohydrate with placebo, low dose and high dose seaweed extracts. Values are means with standard deviation error bars. High dose: 560 mg polyphenols. Low dose: 140 mg polyphenols.

Postprandial curves for blood glucose are illustrated in Figure 2. Descriptive statistics of fasting and postprandial blood glucose are reported in Table 2. Prior to each treatment, there were no differences in fasting blood glucose levels (Wilk’s Lambda = 0.902, F = 1.950, *p* = 0.157, multivariate partial eta squared = 0.098). There were also no differences in postprandial blood glucose iAUC (Wilk’s Lambda = 0.986, F = 0.227, *p* = 0.798, multivariate partial eta squared = 0.014) or peak concentration (Wilk’s Lambda = 0.963, F = 0.601, *p* = 0.555, multivariate partial eta squared = 0.037) between the placebo, low dose, or high dose treatments. There were also no differences between those of Asian and non-Asian backgrounds, across all three treatments, for postprandial blood glucose iAUC (*p* = 0.208, partial eta squared = 0.044) or peak blood glucose concentration (*p* = 0.393, partial eta squared = 0.020) (Table 2). Supplement sequence (*p* = 0.706, 0.997), age (*p* = 0.689, 0.658), gender (*p* = 0.446, 0.329), and percent fat mass (*p* = 0.815, 0.555) did not influence the effect of the brown seaweed extract on postprandial blood glucose iAUC or peak concentration, respectively. Mean differences between the treatments are presented in Supplementary Table S1. Blood glucose time to peak data are presented in Supplementary Table S2.

### 3.3. Postprandial Plasma Insulin

**Figure 3:**
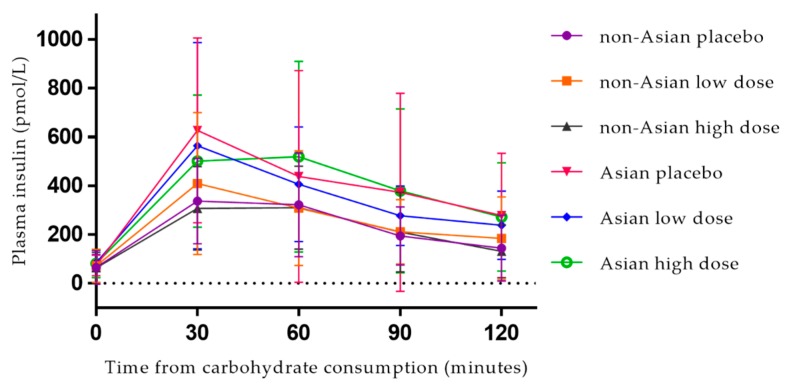
Postprandial plasma insulin response for non-Asian (*n* = 26) and Asian (*n* = 12) participants following consumption of 50 g available carbohydrate with placebo, low-dose, and high-dose seaweed extracts. Values are means with standard deviation error bars. High dose: 560 mg polyphenols. Low dose: 140 mg polyphenols.

Postprandial curves for plasma insulin are illustrated in Figure 3. Descriptive statistics for fasting and postprandial plasma insulin are reported in Table 2. Prior to each treatment, there were no differences in fasting plasma insulin levels (Wilk’s Lambda = 0.936, F = 1.238, *p* = 0.302, multivariate partial eta squared = 0.064). There were no differences in postprandial plasma insulin iAUC (Wilk’s Lambda = 0.995, F = 0.084, *p* = 0.920, multivariate partial eta squared = 0.005) or peak concentration (Wilk’s Lambda = 0.883, F = 2.054, *p* = 0.145, multivariate partial eta squared = 0.117) between the placebo, low dose or high dose treatments. Supplement sequence (*p* = 0.516, 0.191), age (*p* = 0.426, 0.356), gender (*p* = 0.731, 553) and percent fat mass (*p* = 0.710, 376) did not influence the effect of the brown seaweed extract on postprandial plasma insulin iAUC or peak concentration, respectively. 

**Figure 4:**
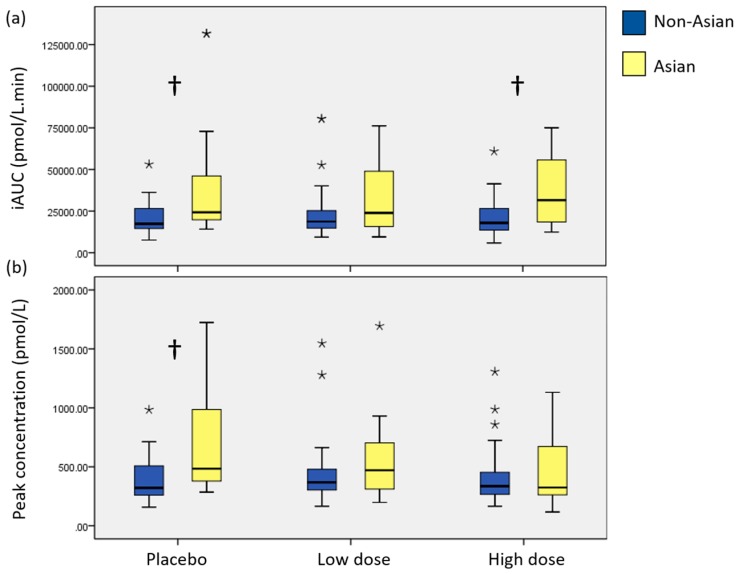
Plasma insulin postprandial incremental area under the curve (**a**) and peak concentration (**b**) results following placebo, low-dose, and high-dose seaweed extracts, comparing those of non-Asian (*n* = 26, blue) and Asian (*n* = 12, yellow) backgrounds; † difference between ethnic groups, *p* ≤ 0.01. * shows outliers on graphs. High dose: 560 mg polyphenols. Low dose: 140 mg polyphenols.

Across all treatments, participants of an Asian background had a significantly higher postprandial plasma insulin iAUC (*p* = 0.016, partial eta squared = 0.180). Peak concentration was higher among Asian participants, but this did not reach significance (*p* = 0.065, partial eta squared = 0.109). Further investigation, by independent *t*-tests, identified that differences between Asian and non-Asian participants for plasma insulin iAUC were present following the placebo and high dose treatments (*p* = 0.010 and 0.006, respectively) and in peak insulin concentration following placebo only (*p* = 0.010) (Figure 4). There were no differences between Asian and non-Asian participants for fasting plasma insulin levels. Mean differences between groups are presented in Supplementary Table S1. Plasma insulin time to peak data are presented Supplementary Table S2.

### 3.4. Intolerance Symptoms

The four intolerance symptoms reported most frequently were tiredness/exhaustion, lack of energy, headache, and abdominal bloating. These symptoms were reported by participants across all three treatment arms. The majority of symptoms were reported to be of mild intensity (1 on an arbitrary scale of 0 to 3). There were no differences between the groups for any of the symptoms assessed (data not shown).

### 3.5. Phlorotannin Contents

The total soluble phlorotannin concentration of the extract was assessed by the Folin-Ciocalteu method in water extracts of powdered *Fucus vesiculosus*. The phlorotannin concentration in the extract was 29.7% dry weight, according to the phloroglucinol standard curve. The mean coefficient of variation among the triplicates was 2.5%. This is comparable to the suggested 28% polyphenol content in the extract, as certified by the supplier.

## 4. Discussion

The aim of this study was to investigate the impact of two doses (500 mg and 2000 mg) of a polyphenol-rich seaweed extract, compared with placebo, on postprandial glycaemic responses in healthy adults. Across the three treatments, there were no differences in the iAUC or postprandial peak concentrations for blood glucose or plasma insulin. These findings indicate that in healthy adults, a polyphenol-rich seaweed extract has no additional lowering effects on glycaemic response over that of two grams of cellulose fibre.

These results are in contrast with the original hypothesis, which was based on evidence for the glycaemic lowering effects of marine polyphenols [10,17]. The mechanism by which polyphenols are understood to reduce postprandial glycaemic responses is through the inhibition of carbohydrate digesting enzymes α-amylase and α-glucosidase. This action has been demonstrated in vitro using polyphenols from a variety of marine algal sources, including *Fucus vesiculosus* [18,19,20,21,22]. This effect has also been demonstrated in mice, where mice who received polyphenols had reduced postprandial blood glucose levels compared with control mice [19,20,22]. However, when this type of intervention has been translated into humans, the results from the only two previous studies were inconsistent [26]. 

Lee and Jeon [23] conducted a 12-week intervention trial in individuals with high fasting blood glucose in Korea, where 2-h postprandial blood glucose was measured at baseline and week 12 in both the intervention and placebo groups. Their study design was quite different to the present study, being a long-term intervention study as opposed to a one-off postprandial test. They also used a different algal species (*Ecklonia cava*) and specified that their extract was rich in the phlorotannin dieckol; however, the dose of polyphenols given (690 mg/day) was similar to the high-dose supplement in this study (560 mg). Unlike the present study, they found that 2-h postprandial blood glucose levels reduced significantly in the intervention group after taking the polyphenol-rich extract for 12 weeks, compared with no change in the placebo group. Paradis et al. [24] conducted a crossover trial in healthy individuals in Canada, comparing the effects of an extract blended from *Fucus vesiculosus* and *Ascophyllum nodosum* (containing 25 mg unspecified polyphenols) with a placebo (containing 287 mg calcium phosphate basic and 191 mg microcrystalline cellulose), on 3-h postprandial blood glucose and insulin. Their study design was similar to the present study, with the polyphenol supplement ingested 30 min prior to 50 g available carbohydrate from white bread (to allow time for the polyphenols to reach the intestine), followed by repeated postprandial blood sampling. However, they used a much lower dose of polyphenols (25 mg). Similarly, their results indicated no effect of the intervention on postprandial blood glucose iAUC; however, they found a reduction in iAUC for plasma insulin following the intervention, which was not seen in the present study. Overall, results remain inconsistent. However, the evidence to date would suggest that longer-term consumption might be necessary to see a glucose lowering effect, as opposed to a single dose. It also suggests that polyphenol supplementation may only be effective in populations with dysregulated glucose control, as opposed to healthy populations.

Similar findings have been observed when the effects of polyphenols from land-based sources on postprandial glycaemia were investigated. A recent review by Coe and Ryan [17] found mixed effects on postprandial peak concentrations and iAUC for glucose and insulin among healthy individuals. They concluded that polyphenols, when consumed with carbohydrates, had the greatest lowering effect on peak and early-phase blood glucose response. However, it was noted that the degree of glycaemic lowering effects was dependent on the source of polyphenols, the dose used, and the composition of the polyphenols used [17]. These factors were also highlighted by a recent review of marine algal-based polyphenol interventions; however, due to the small number of studies in this area, it is difficult to conclude how factors such as dose and polyphenol source influenced the effect of the intervention on glycaemic response [26]. 

The present study is the first to examine the effects of polyphenols solely from *Fucus vesiculosus* on glycaemic responses in humans. However, Paradis et al. [24], as discussed above, investigated a polyphenol-rich extract prepared from a blend of *Ascophyllum nodosum* and *Fucus vesiculosus*, which showed a lowering effect on postprandial insulin. Polyphenols from *Fucus vesiculosus* have also successfully demonstrated digestive enzyme inhibition in vitro and delayed postprandial blood glucose responses in rats [22]. In regard to the doses used in present study (140 mg and 560 mg polyphenols), they are similar to polyphenol doses used in previous human studies (144 mg [29] and 690 mg [23]) that have shown efficacy in improving other biochemical markers (e.g., LDL cholesterol, fasting blood glucose) [22,23,29]. Therefore, it is unlikely that the lack of effect observed in the present study was due to an inadequate dose or the source of the polyphenols. However, the effects of human gastrointestinal digestion and absorption on the polyphenols must be considered—in particular, any changes in structure or concentration that occur when the compounds enter the human body that may affect their biological activity [39,40]. These issues highlight the need for further investigations, to move away from in vitro work and animal models and into human populations.

Our choice of placebo (cellulose fibre) was to match the complex carbohydrate component (fucoidan) of the algal extract. However, at only 67% of the supplement, there was a discrepancy in fibre content between the active supplement and placebo, with one third of the marine extract comprising the polyphenol component. It is important to note that fibre has a lowering effect on postprandial glycaemia [41,42] and could be a potential reason for the lack of difference between the placebo and supplement in terms of glycaemic response. More likely, however, is that the population selected for this trial was healthy adults with already well-controlled glycaemic responses. As such, the polyphenols have shown no additional glycaemic lowering effects over that of the placebo. While the comparison of polyphenols to a fibre is valuable to assess the efficacy of polyphenols at reducing glycaemic responses, future research may also consider comparing seaweed extracts to both fibre and non-fibre placebos, to evaluate the glycaemic lowering effects of seaweed extracts as a whole, given their potential for commercialization as an alternative source of fibre [43]. 

A potential explanation for the lack of impact of the polyphenol-rich supplement on glycaemic response is that the population of this study consisted of healthy individuals. Participants had normal fasting blood glucose levels (<5.5 mmol/L) and presumably optimal glucose tolerance. A healthy population was selected for this study to first investigate dosing, with an aim to trial the novel supplement in an at-risk population next, if effective. However, it appears that the supplement caused no improvement in glycaemic control in the already-healthy volunteers. In the two studies that previously looked at the effects of polyphenol-rich seaweed extracts on postprandial glycaemic responses, Paradis et al. [24] examined non-diabetic, healthy individuals, and saw no effect on postprandial blood glucose response. However, Lee and Jeon [23], who assessed individuals with high fasting blood glucose levels (between 100 and 180 mg/dL), observed an improvement in postprandial blood glucose control following treatment. This indicates that supplementation with a polyphenol-rich seaweed extract may only improve postprandial blood glucose responses when they are not already effectively regulated. This finding suggests that further research should target individuals with impaired fasting glucose or impaired glucose tolerance. There is also emerging, yet consistent evidence that healthy people experience elevated postprandial blood glucose responses when consuming carbohydrates in the evening and at night, similar to that of individuals with impaired glucose tolerance [44,45,46,47]. The effect of timing, in particular giving a supplement in the evening, is another area in which the anti-hyperglycaemic effects of a polyphenol-rich seaweed extract on could be investigated, to potentially reduce the long-term risk of T2DM [46,48].

A secondary outcome of this research was to identify whether there was a difference in the effect of the polyphenol-rich seaweed extract on postprandial glycaemic response between those of non-Asian and Asian backgrounds. People of Asian descent who have adopted a Western lifestyle experience an increased risk of T2DM in comparison to their Caucasian counterparts [2,3]. In 2001, the prevalence of diabetes among Southeastern- and Southern Asian-born Australians was 1.9 and 1.5 times higher than Australian-born males and females, respectively [49]. It has been suggested that this increased risk may be due to an increased prevalence of insulin resistance among Asian populations, which in turn has been linked to an accumulation of visceral adipose tissue [50,51]. This is consistent with our findings of a higher postprandial insulin response in individuals of an Asian background compared with those of a non-Asian background, despite no differences in glucose tolerance. As well as significantly greater amounts of visceral adipose tissue among Asian participants than non-Asian participants. However, the differences in insulin response between the ethnic backgrounds were following the placebo and high-dose treatments, which contained 2000 mg and 1200 mg of fibre, respectively. This suggests either that the elevated insulin response experienced by individuals of Asian descent could not be attenuated by a single dose of a polyphenol-rich extract or a 2 g fibre supplement, or, that individuals of a non-Asian background had a greater response to the fibre or polyphenol content of these supplements than individuals of Asian descent, resulting in lower insulin responses. Either way, these differences need to be taken into account when designing future research studies with ethnically diverse populations or in different countries.

Overall, these results indicated that in young, healthy Asian people, reduced insulin sensitivity can be present without any sign of impaired fasting glucose or impaired glucose tolerance. Therefore, glucose tolerance tests alone may be insufficient to diagnose T2DM risk in Asian populations. Further investigation into the most appropriate way to diagnose T2DM risk among Asian populations is required, and should include an assessment of both glucose and insulin levels. 

## 5. Conclusions

This study indicated that a single dose of up to 2000 mg of a polyphenol-rich *Fucus vesiculosus* extract has no additional lowering effect over that of two grams of cellulose fibre on postprandial blood glucose or plasma insulin in healthy adults. Further research should investigate the glycaemic modulating effects of polyphenol-rich marine algal extracts in at-risk populations, such as pre-diabetics, as this is where effects have been identified to date. This study also identified that participants of an Asian background had elevated postprandial insulin responses, even with normal blood glucose levels, indicative of reduced insulin sensitivity and an increased risk of developing T2DM [50,52]. A new method for assessing T2DM risk in Asian populations is needed, as oral glucose tolerance tests fail to identify reduced insulin sensitivity where glucose tolerance is not affected. A new method of assessment should include measures of both fasting and postprandial glucose and insulin levels.

## Figures and Tables

**Table 1:** Participant characteristics (*n* = 38).

Characteristic	Total Population	Non-Asian Background	Asian Background
	**Median (IQR ^1^)**	**Median (IQR)**	**Median (IQR)**
Age (years)	23 (11)	24 (17)	21 (5)
BMI (kg/m^2^)	21.9 (3.0)	23.0 (2.3)	21.0 (1.9)
Waist circumference (cm)	72.0 (8.3)	73.0 (7.5)	68.5 (12.5)
Total fat mass (%)—females	27.6 (4.7)	27.3 (4.8)	27.6 (7.8)
Total fat mass (%)—males	13.9 (7.5)	13.4 (7.1)	14.1 (13.2 *)
Estimated daily polyphenol intake (mg)	801 (732)	930 (561)	375 (362)
Estimated daily energy intake (kJ)	9443 (4233)	9214 (4150)	9739 (7427)
Systolic blood pressure (mmHg)	121 (15)	125 (13)	115 (11)
Diastolic blood pressure (mmHg)	76 (7)	76 (8)	77 (7)
Visceral fat (L)	0.6 (0.8)	0.3 (0.5)	1.1 (0.4)
	***n* (frequency)**	***n* (frequency)**	***n* (frequency)**
Female	29 (76%)	20 (77%)	9 (75%)
Male	9 (24%)	6 (23%)	3 (25%)
**Physical Activity level**			
Low	6 (16%)	5 (19%)	1 (8%)
Moderate	10 (26%)	3 (12%)	7 (58%)
High	22 (58%)	18 (69%)	4 (34%)

^1^ IQR—Interquartile range; * median (range) reported due to inadequate participant numbers for IQR.

**Table 2:** Fasting and postprandial blood glucose and plasma insulin measures, for placebo, low-dose, and high-dose seaweed extracts, according to ethnic background.

Outcome	Ethnic Background	*n*	Placebo	Low Dose	High Dose	*p* (Effect of Supplement)	*p* (Effect of Ethnicity)
**Blood glucose**							
Fasting (mmol/L)	Total	38	4.4 (0.3)	4.5 (0.4)	4.4 (0.4)	0.157	NA
	non-Asian	26	4.3 (0.4)	4.5 (0.5)	4.4 (0.5)	0.154	0.787
	Asian	12	4.4 (0.3)	4.5 (0.3)	4.5 (0.4)	0.914	
iAUC (mmol/L·min)	Total	38	151.9 (61.1)	136.2 (57.5)	145.7 (62.8)	0.798	NA
	non-Asian	26	145.6 (61.3)	129.8 (63.5)	136.3 (62.3)	0.168	0.208
	Asian	12	165.6 (61.0)	150.2 (40.7)	166.1 (61.6)	0.972	
Peak concentration (mmol/L)	Total	38	6.9 (0.8)	6.9 (0.8)	6.9 (0.8)	0.555	NA
	non-Asian	26	6.8 (0.7)	6.9 (0.8)	6.8 (0.8)	0.402	0.393
	Asian	12	7.1 (1.0)	6.9 (0.9)	7.1 (0.8)	0.602	
**Plasma insulin**							
Fasting (pmol/L) ^a^	Total	38	53.5 (54.0)	71.7 (50.2)	56.5 (57.3)	0.302	NA
	non-Asian	26	50.9 (38.8)	69.6 (55.5)	52.1 (55.2)	0.721	0.198
	Asian	12	73.2 (85.1)	80.6 (50.1)	64.1 (73.8)	0.103	
iAUC (pmol/L·min) ^a^	Total	38	19,451 (16722)	20,421 (13207)	19,967 (15917)	0.920	NA
	non-Asian	26	17,380 (13357) ^b^	18,702 (11095)	17,874 (13279) ^b^	0.278	0.016
	Asian	12	24,231 (28205) ^b^	23,923 (34482)	31,573 (40005) ^b^	0.306	
Peak concentration (pmol/L) ^a^	Total	38	382.5 (367.7)	373.3 (257.1)	325.8 (305.1)	0.145	NA
	non-Asian	26	321.6(279.8) ^b^	368.9 (180.9)	336.1 (189.5)	0.755	0.065
	Asian	12	484.1 (637.8) ^b^	471.3 (413.7)	324.5 (433.8)	0.158	

^1^ All parametric values reported as mean (standard deviation); ^a^ non-parametric data reported as median (interquartile range); ^b^ difference between ethnic groups with *p* ≤ 0.01. High dose: 560 mg polyphenols. Low dose: 140 mg polyphenols.

## Supplementary Materials

The following are available online at http://www.mdpi.com/2072-6643/10/3/270/s1, Table S1: Mean differences in blood glucose and plasma insulin iAUC and peak concentrations between placebo and low dose, placebo, and high dose, and low dose and high dose, with groupings according to ethnic background, Table S2: Blood glucose and plasma insulin time to reach peak postprandial concentration (*n* = 38).
