# Supplementary material for: The Impact of a Single Dose of a Polyphenol-Rich Seaweed Extract on Postprandial Glycaemic Control in Healthy Adults: A Randomised Cross-Over Trial
Source: Nutrients. 2018 Feb 27;10(3):270. doi: 10.3390/nu10030270 (PMC5872688; doi:10.3390/nu10030270)
Supplement: Supplementary file 1 [file nutrients-10-00270-s001.zip › Table S1 mean differences.docx]

**Table S1:** Mean differences in blood glucose and plasma insulin iAUC and peak concentrations between placebo and low dose, placebo and high dose, and low dose and high dose, with groupings according to ethnic background.

| **Blood glucose iAUC (mmol/L.min)** | | | | |
| --- | --- | --- | --- | --- |
| **Ethnicity** | **Mean differences** | **n** | **Mean** | **SD** |
| **Total** | Low dose - placebo | 38 | -16.2 | 56.4 |
|  | High dose - placebo | 38 | -6.5 | 48.9 |
|  | High dose - low dose | 38 | 9.7 | 49.4 |
| **Non-Asian** | Low dose - placebo | 26 | -16.9 | 55.5 |
|  | High dose - placebo | 26 | -10.0 | 42.5 |
|  | High dose - low dose | 26 | 6.8 | 42.3 |
| **Asian** | Low dose - placebo | 12 | -14.8 | 60.7 |
|  | High dose - placebo | 12 | 1.2 | 62.1 |
|  | High dose - low dose | 12 | 16.0 | 64.0 |
| **Blood glucose peak concentration (mmol/L)** | | | | |
| **Ethnicity** | **Mean differences** | **n** | **Mean** | **SD** |
| **Total** | Low dose - placebo | 38 | -0.03 | 0.87 |
|  | High dose - placebo | 38 | 0.02 | 0.74 |
|  | High dose - low dose | 38 | 0.05 | 0.78 |
| **Non-Asian** | Low dose - placebo | 26 | 0.04 | 0.79 |
|  | High dose - placebo | 26 | 0.03 | 0.60 |
|  | High dose - low dose | 26 | -0.01 | 0.80 |
| **Asian** | Low dose - placebo | 12 | -0.19 | 1.05 |
|  | High dose - placebo | 12 | -0.02 | 1.00 |
|  | High dose - low dose | 12 | 0.18 | 0.75 |
| **Plasma insulin iAUC (pmol/L.min)** | | | | |
| **Ethnicity** | **Mean differences** | **n** | **Median** | **IQR** |
| **Total** | Low dose - placebo | 38 | -819 | 10589 |
|  | High dose - placebo | 38 | -1340 | 11674 |
|  | High dose - low dose | 38 | -583 | 8326 |
| **Non-Asian** | Low dose - placebo | 26 | -396 | 9604 |
|  | High dose - placebo | 26 | -1340 | 10424 |
|  | High dose - low dose | 26 | -1333 | 6472 |
| **Asian** | Low dose - placebo | 12 | -2278 | 11639 |
|  | High dose - placebo | 12 | -1382 | 15667 |
|  | High dose - low dose | 12 | 4271 | 13833 |
| **Plasma insulin peak concentration (pmol/L)** | | | | |
| **Ethnicity** | **Mean differences** | **n** | **Median** | **IQR** |
| **Total** | Low dose - placebo | 38 | -28.5 | 184.0 |
|  | High dose - placebo | 38 | 2.8 | 126.4 |
|  | High dose - low dose | 38 | -9.7 | 176.4 |
| **Non-Asian** | Low dose - placebo | 38 | -15.3 | 181.9 |
|  | High dose - placebo | 38 | -2.8 | 109.0 |
|  | High dose - low dose | 38 | -54.9 | 144.4 |
| **Asian** | Low dose - placebo | 26 | -83.3 | 154.9 |
|  | High dose - placebo | 26 | 18.8 | 209.7 |
|  | High dose - low dose | 26 | 80.6 | 158.3 |

iAUC – incremental area under the curve
