# Supplementary material for: The Impact of a Single Dose of a Polyphenol-Rich Seaweed Extract on Postprandial Glycaemic Control in Healthy Adults: A Randomised Cross-Over Trial
Source: Nutrients. 2018 Feb 27;10(3):270. doi: 10.3390/nu10030270 (PMC5872688; doi:10.3390/nu10030270)
Supplement: Supplementary file 1 [file nutrients-10-00270-s001.zip › Table S2 Time to peak table.docx]

**Table S2**: Blood glucose and plasma insulin time to reach peak postprandial concentration, n=38

| **Supplement** | **Time point (minutes)** | | | | |
| --- | --- | --- | --- | --- | --- |
|  | **30** | **45** | **60** | **90** | **120** |
| **Blood glucose** | | | | | |
| **Placebo** | 16 (42%) | 19 (50%) | 3 (8%) | 0 (0%) | 0 (0%) |
| **Low dose** | 13 (34%) | 19 (50%) | 3 (8%) | 1 (3%) | 2 (5%) |
| **High dose** | 12 (32%) | 21 (55%) | 5 (13%) | 0 (0%) | 0 (0%) |
| **Plasma insulin** | | | | | |
| **Placebo** | 24 (63%) |  | 13 (34%) | 0 (0%) | 1 (3%) |
| **Low dose** | 26 (68%) |  | 10 (26%) | 2 (5%) | 0 (0%) |
| **High dose** | 19 (50%) |  | 16 (42%) | 3 (8%) | 0 (0%) |

Data presented as n (%)
